# Comparison of the Peel-Associated Epiphytic Bacteria of Anthocyanin-Rich “Sun Black” and Wild-Type Tomatoes under Organic and Conventional Farming

**DOI:** 10.3390/microorganisms10112240

**Published:** 2022-11-12

**Authors:** Susanna Gorrasi, Marcella Pasqualetti, Barbara Muñoz-Palazon, Giorgia Novello, Andrea Mazzucato, Enio Campiglia, Massimiliano Fenice

**Affiliations:** 1 Department of Ecological and Biological Sciences (DEB), University of Tuscia, Largo Università snc, 01100 Viterbo, Italy; 2 Laboratory of Ecology of Marine Fungi, CoNISMa, Department of Ecological and Biological Sciences, University of Tuscia, Largo Università snc, 01100 Viterbo, Italy; 3 Institute of Water Research, University of Granada, 18071 Granada, Spain; 4 Department of Science, Technology and Innovation (DISIT), Università del Piemonte Orientale, Viale Teresa Michel, 11, 15121 Alessandria, Italy; 5 Department of Agricultural and Forest Sciences (DAFNE), University of Tuscia, Via San Camillo de Lellis snc, 01100 Viterbo, Italy; 6 Laboratory of Applied Marine Microbiology, CoNISMa, University of Tuscia, Largo Università snc, 01100 Viterbo, Italy

**Keywords:** *Solanum lycopersicum* L., tomato, purple tomato, anthocyanin-rich tomato, sun black tomato, organic farming, conventional farming, bacterial communities, epiphytic bacteria, amplicon sequencing

## Abstract

Tomatoes are among the most consumed vegetables worldwide and represent a source of health-beneficial substances. Our study represents the first investigating the peel-associated epiphytic bacteria of red and purple (anthocyanin-rich) tomatoes subjected to organic and conventional farming systems. *Proteobacteria* was the dominant phylum (relative abundances 79–91%) in all experimental conditions. *Enterobacteriaceae* represented a large fraction (39.3–47.5%) of the communities, with *Buttiauxella* and *Atlantibacter* as the most represented genera. The core microbiota was composed of 59 operational taxonomic units (OTUs), including the majority of the most abundant ones. The occurrence of the most abundant OTUs differed among the experimental conditions. OTU 1 (*Buttiauxella*), OTU 2 (*Enterobacteriales*), and OTU 6 (*Bacillales*) were higher in red and purple tomatoes grown under organic farming. OTU 5 (*Acinetobacter*) had the highest abundance in red tomatoes subjected to organic farming. OTU 3 (*Atlantibacter*) was among the major OTUs in red tomatoes under both farming conditions. OTU 7 (*Clavibacter*) and OTU 8 (*Enterobacteriaceae*) had abundances ≥1% only in red tomatoes grown under conventional farming. PCA and clustering analysis highlighted a high similarity between the bacterial communities of red and purple tomatoes grown under organic farming. Furthermore, the bacterial communities of purple tomatoes grown under organic farming showed the lowest diversity and evenness. This work paves the way to understand the role of nutritional superior tomato genotypes, combined with organic farming, to modulate the presence of beneficial/harmful bacteria and supply healthier foods within a sustainable agriculture.

## 1. Introduction

The tomato (*Solanum lycopersicum* L.) represents an economically important vegetable crop, with a world production of over 186 million tons [1]. It is one of the most consumed vegetables in the world and a common food in the Mediterranean diet, and it is produced and consumed both as fresh and processed products (e.g., paste, peeled tomatoes, diced products, and sauces) [2].

Tomatoes are a dietary source of health-beneficial substances, such as fibers, vitamins, minerals, and antioxidants (e.g., phenolic compounds, β-carotene, and in particular lycopene) [3,4]. Due to its nutritional importance and to an increasing consumers’ interest in food production and quality, high attention has been paid both to sustainable agricultural practices and to breeding strategies aiming at improving the shelf-life, the quality traits, and the nutraceutical value of tomato (e.g., high content in phytochemicals and bio-active compounds) [5,6].

In recent decades, there has been a worldwide awareness increase about the need of sustainable agricultural practices, especially focused to increase yield while also addressing environmental conservation and human health. Sustainable cropping systems are intended to preserve soil health and improve its chemical-physical characteristics, reduce synthetic fertilizer and pesticide inputs, and increase product quality [7].

The techniques used to increase tomato yield, paying attention to environmental sustainability and human health, include the hairy vetch mulch system, the use of plant-beneficial microorganisms to improve the fruit quality and reduce fertilization, the development of new cultivars with enhanced nutritional qualities and tolerance/resistance to abiotic and biotic stresses [8,9,10].

The development of nutritionally superior genotypes, in particular the selection of cultivars having high contents of antioxidants (such as phenolic compounds), is becoming a significant research trend. Among them, anthocyanins are secondary metabolites of the flavonoid family, playing a significant role in plant tolerance to biotic/abiotic stress and in the prevention and treatment of various important human diseases [11,12].

Several studies based on both conventional breeding and genetic engineering strategies have allowed obtaining anthocyanin-rich tomato lines [11]. These dark-skinned colored tomatoes (so-called “purple” or “black” tomatoes) result from mutations in carotenoid/flavonoid biosynthetic pathways [13]. Compared to the wild type, tomatoes enriched in anthocyanins have shown higher antioxidant activity and increased resistance to some pathogens, resulting in a significant extension of the shelf life [14]. 

A 20-year breeding activity carried out at the University of Tuscia (Viterbo, Italy) allowed to select and fix the anthocyanin-rich tomato line called “Sun Black” (SB). It was obtained by combining the mutations of *Anthocyanin fruit* (*Aft*) and *atroviolaceum* (*atv*) alleles, affecting the anthocyanin biosynthesis, in the same line [15]. The SB tomato features an accumulation of anthocyanins in the peel and other bioactive compounds (e.g., gentistic acid, rutin, and vitamin C) in the whole fruit [13].

The studies carried out on purple tomatoes have been mainly focused on the characterization of their nutritional/bioactive compounds and health beneficial effects and, to a lesser extent, on their longer shelf life and resistance to some pathogens [13,14,15,16]. 

Phyllospheric microbiota refers to the microorganisms found on the above-ground part of the plants (e.g., stems, leaves, flowers, and fruits), which may be epiphytic and endophytic, and can be both beneficial or harmful to the host plant [17,18]. Actually, investigating the epiphytic microbial communities of fruits and vegetables is important to improve food quality and safety, being the consumers directly exposed to these microorganisms. This is particularly true for widely consumed fresh products. In fact, fresh fruits and vegetables have been recognized as reservoirs of foodborne pathogens and spoilage microorganisms [19,20]. On the other hand, the microbiota naturally present on these foodstuffs harbors also beneficial representatives, which are involved in plant fitness and produce a number of industrially important compounds (e.g., antibiotics, enzymes, organic acids, and vitamins) [21,22,23]. Among them, the bioactive compound producers (having antagonistic effects on plant pathogens) can be employed as biological control agents [18,24,25]. Although the plant episphere, besides being a reservoir of possible harmful microorganisms, has been proven to be an important source of beneficial microorganisms (and their bioactive compounds), epiphytic microbial communities are the least investigated among the plant microbiota.

To the best of our knowledge, in general, no study has compared the microbiota of red and purple tomatoes, particularly in relation to peel-associated epiphytic bacteria. Furthermore, the bacterial diversity harbored by two genotypes subjected to different farming practices has never been studied. Since microorganisms are known to be involved in ecosystem functioning, plant physiology and response to abiotic/biotic stresses, as well as in phytopathology and infectious disease outbreaks, a focus on the two tomato phyllospheric microbiota would be of scientific and economic interest.

This work aimed to characterize the peel-associated epiphytic bacteria of SB and the near-isogenic wild type (WT) tomatoes grown under two farming systems (organic and conventional), shedding light on possible assemblage differences in their bacterial communities. This would provide useful information on the importance of combining nutritional superior tomato genotypes with the right farming management, to provide healthier foods in a sustainable agriculture framework.

## 2. Materials and Methods

### 2.1. Plant Materials and Growth Conditions

The research was carried out on the two tomato lines (SB and WT) grown in open field at the Experimental Farm “Nello Lupori” (42°25′35.71818″ N; 12°4′49.34524″ E) of the University of Tuscia (Viterbo, Italy) under organic and conventional farming (referred here as OF and CF, respectively). The two agrotechnical protocols, differing in the precession crop and soil management, were applied as previously reported [26,27]. The experimental conditions (genotype × farming condition) considered in this study were: T1, WT/OF; T2, WT/CF; T3, SB/OF; T4, SB/CF.

The two lines were previously selected by Mazzucato et al. [15] and, except for the different fruit colors (SB and WT are purple- and red-colored, respectively), they are characterized by the same phenotypic traits. 

For both lines, seeds were germinated in Petri dishes, then plantlets were transplanted in twin rows (planting distances: 160 cm between twins, 40 cm between rows, and 50 cm between plants within the row) at the 4–5th true leaf stage. Ten plants per line were arranged in twin rows using a randomized block design with three replicates. Plants were grown on tutors with the standard agronomic practices for fresh market tomatoes. Lateral shoots were weekly removed, and plants were left to open pollination.

The soil in the experimental area was a Typic Xerofluvent (volcanic) with the following characteristics in the 0–30 cm layer: 668 g kg^−1^ of dry soil sand, 194 g kg^−1^ of dry soil silt, 138 g kg^−1^ of dry soil clay, bulk density 1.40 g cm^−3^, pH 6.43 (water, 1:2.5), organic matter 17.5 g kg^−1^ of dry soil, total nitrogen 1.01 g kg^−1^ of dry soil, and available phosphate 13.92 g kg^−1^ of dry soil.

### 2.2. Sample Collection and Processing

For each experimental condition (genotype × farming), a total of 16 healthy and representative fruits (composite sample) were aseptically sampled (they were collected wearing gloves and put into sterile plastic bags) from the three crop replicates. To obtain the composite sample, four fruits were collected from four plants located in different crop replicates. The fruits were taken from various positions on the plant, avoiding those showing the skin covered by soil debris [28]. The samples were immediately transported to the nearby laboratory to be processed. 

The harvested fruits were softly pre-washed to remove debris and microorganisms that may have been incidentally deposited on the fruit surface, as reported by Janisiewicz et al. [29] with some adjustments: each sample was transferred to a sterile plastic bag with 1000 mL of sterile deionized water and gently hand massaged for 1 min. The pre-washing water was discarded and the recovery of the skin-associated microorganisms was performed as reported by Fernández-Suárez et al. [30] with slight modifications. The washing solution (1 L of sterile water containing 0.1% Tween 20) was added to the bag that was closed and placed into another bag. The double-bagged fruits were gently shaken for 45 min. The above washings were carried out 25 °C. The washing solutions were vacuum-filtered on sterile 0.22 µm nitrocellulose membranes (Millipore, Burlington, MA, USA), which were kept frozen until DNA extraction.

### 2.3. DNA Extraction, 16S rRNA Gene Amplicon Libraries and Sequencing

Total DNA was extracted from filters using the ZR Fungal/Bacterial DNA MiniPrep kit (Zymo Research Corp., Irvine, CA, USA) following the manufacturer’s instructions. A two-step PCR was used to prepare the multiplexed amplicon libraries. To profile the peel-associated bacterial diversity, the V5–V6 hypervariable regions of the 16S rRNA gene were amplified using the primer set 783F/1046R [31,32]. The strategy and the protocols used to prepare the libraries have already been described in detail by Gorrasi et al. [33]. The amplicon libraries were sequenced at Nuova Genetica Italiana by Illumina MiSeq (Illumina, San Diego, CA, USA) using a 2 × 250 bp paired-end protocol. 

The sequence data have been submitted to the Sequence Read Archive (SRA) database under the accession number PRJNA798869.

### 2.4. Sequence Processing and Data Analysis

Sequence processing and data analyses were performed as previously reported [34,35]. 

Raw reads were demultiplexed according to the indices and internal barcodes and processed using the mothur software version 1.44.3. Forward and reverse reads were merged into contigs with a quality score of mismatched base = 0. The reads were subjected to quality trimming to remove sequences with more than eight homopolymers and ambiguous bases. The suspected chimeras were identified using the VSEARCH algorithm and then removed [35]. Unique sequences were determined and then mapped against the original dataset to calculate the abundance data. The high-quality reads were clustered into operational taxonomic units (OTUs) using a 97% similarity cut-off. Singletons (sequences appearing only once in the entire dataset) were removed. 

Taxonomic profiling was assessed using the RDP classifier [36], applying a 50% confidence cut-off for the assignation [37].

### 2.5. Statistical Methods

Alpha diversity was described by the Shannon diversity and Pielou evenness indices. The Shannon index ranges from 0 to +∞ and increases as the community richness and evenness increase [38]. Pielou evenness index ranges from 0 to 1, and decreasing values indicate a decrease in evenness and a prevalence of few dominant species [39]. Alpha diversity indices were calculated using Species Diversity & Richness IV (SDR-IV) software (PISCES Conservation Ltd., Lymington, UK), and index comparison among the experimental conditions was assessed with the Solow test (based on 10,000-iterated randomization test) [40,41]. Indices were calculated on samples rarefied to 5299 randomly chosen sequences, corresponding to the minimum number of sequences at a sample [33].

Beta diversity was analyzed by principal component analysis (PCA) and clustering analysis, based on square root transformed data (to avoid rare OTU overweighting). The PCA analysis was performed using the CANOCO v. 5.1 software package (Microcomputer Power, Ithaca, NY, USA). The clustering analysis (complete linkage) was performed based on Bray–Curtis dissimilarity data, applying the similarity profile (SIMPROF) test [42] to individuate distinct bacterial assemblages among the experimental conditions (statistically different at *p* < 0.05; 1000 permutations and 999 simulations). The clustering analysis was run using the software package PRIMER-E v.6.1.18 (Plymouth, UK).

The shared OTUs among the experimental conditions were visualized by a 4-way Venn diagram, generated using the InteractiVenn tool (http://www.interactivenn.net/index2.html, accessed on 1 October 2022) [43].

## 3. Results

### 3.1. Composition of the Epiphytic Peel-Associated Bacteria

Amplicon sequencing generated a total of 69,948 high-quality bacterial sequences, resulting in 10,287 OTUs defined by the clustering process.

Applying a 50% cut-off [37], OTUs were assigned to 10 phyla, 21 classes, 46 orders, 84 families, and 161 genera. Unassigned OTUs ranged in-between 0.6–5.1%, 0.8–6.4%, 4.3–14.7%, 14.8–38.7%, and 44.0–58.8% at phylum, class, order, family, and genus level, respectively. 

**Figure 1:**
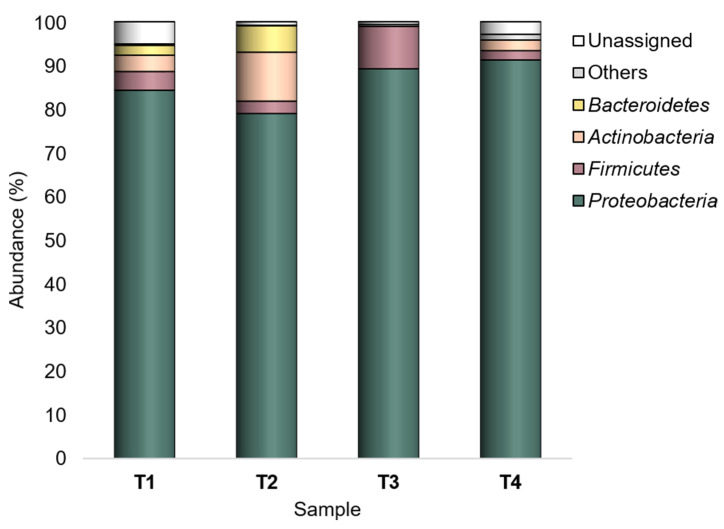
Bacterial composition at the phylum level. Distribution of the major phyla (Ra ≥ 1% in at least one sample); phyla with Ra < 1% were gathered in “Others”. T1: WT/OF, T2: WT/CF, T3: SB/OF, T4: SB/CF.

Overall, the major taxa (having relative abundance (Ra) ≥ 1% in at least one sample) in the peel-associated community were *Proteobacteria*, *Firmicutes*, *Actinobacteria*, and *Bacteroidetes* (Figure 1). Among them, *Proteobacteria* was the dominant phylum (79–91%) in all experimental conditions, with the highest abundance in T4 (SB/CF). *Firmicutes* (Ra_s_ 2.1–9.7%) was found with the highest abundance in T3 (SB/OF). Both *Actinobacteria* and *Bacteroidetes* showed the highest abundances (11.2% and 6% for *Actinobacteria* and *Bacteroidetes*, respectively) in T2 (WT/CF), but they represented minor taxa in some experimental conditions. *Actinobacteria* showed Ra_s_ < 1% in SB tomatoes grown under organic farming (T3), whereas *Bacteroidetes* showed Ra_s_ < 1% in SB tomatoes grown both under organic and conventional farming (T3 and T4).

Overall, the studied peel-associated bacterial flora mostly belonged to the family *Enterobacteriaceae* (39.3–47.5%), being *Buttiauxella* and *Atlantibacter* the most represented genera (with 11,005 and 7156 sequences over the whole dataset, respectively). 

**Figure 2:**
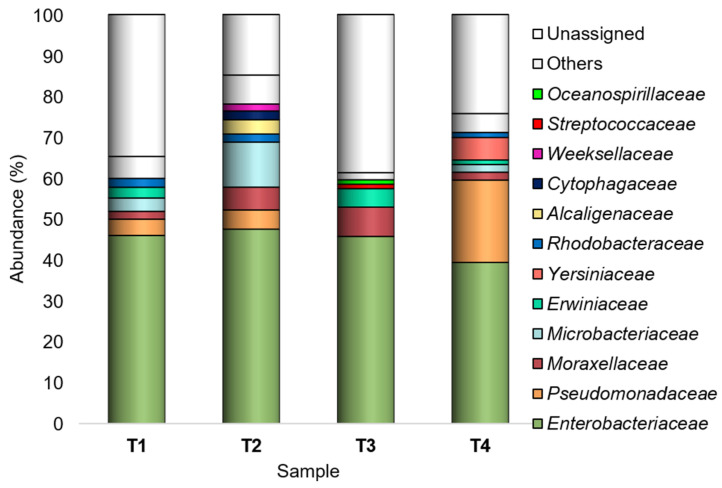
Bacterial composition at the family level. Distribution of the major families (Ra ≥ 1% in at least one sample); families with Ra < 1% were gathered in “Others”. T1: WT/OF, T2: WT/CF, T3: SB/OF, T4: SB/CF.

However, *Enterobacteriaceae*, *Pseudomonadaceae*, *Moraxellaceae*, *Microbacteriaceae*, *Erwiniaceae*, *Yersiniaceae*, *Rhodobacteraceae*, *Alcaligenaceae*, *Cytophagaceae*, *Weeksellaceae*, *Streptococcaceae*, and *Oceanospirillaceae* were recognized as major (Ra ≥ 1% in at least one sample) families (Figure 2).

**Figure 3:**
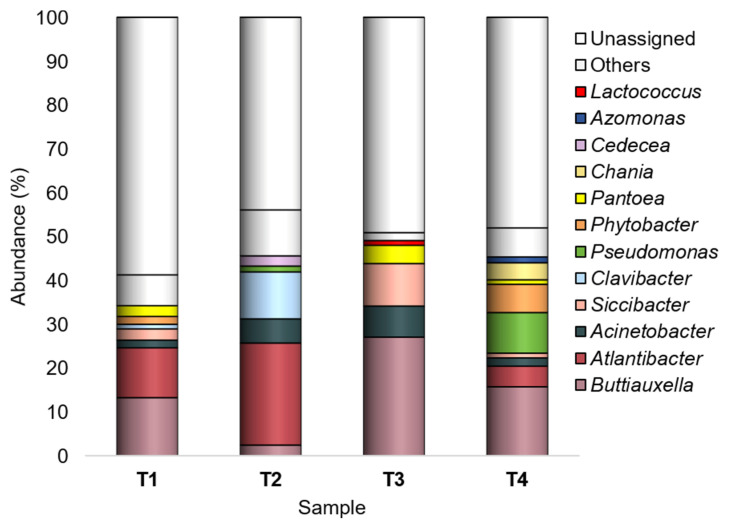
Bacterial composition at the genus level. Distribution of the major genera (Ra ≥ 1% in at least one sample); genera with Ra < 1% were gathered in “Others”. T1: WT/OF, T2: WT/CF, T3: SB/OF, T4: SB/CF.

Among the 161 genera found across all samples, Buttiauxella, Atlantibacter, Acinetobacter, Siccibacter, Clavibacter, Pseudomonas, Phytobacter, Pantoea, Chania, Cedecea, Azomonas, and Lactococcus were recorded as major taxa (Figure 3).

*Buttiauxella* was most abundant in T1, T3, and T4 (Ra_s_ in-between 13.2–27%), with the highest abundances on SB tomato peel; it was least abundant (2.4%) in T2 (WT/CF).

*Atlantibacter* was mainly found in WT tomatoes, having the highest abundance in those grown under CF (23.3%); in SB grown under CF, it was recorded with the lowest abundance (0.7%), representing a rare taxon. 

*Acinetobacter* (Ra_s_ 1.8–7.1%) showed the lowest abundances in T1 and T4 (WT/OF and SB/CF, respectively). 

*Siccibacter*, *Clavibacter*, *Pseudomonas*, and *Phytobacter,* overall recorded with abundances lower than 2.5% and representing sometimes rare taxa, showed a peak in one experimental condition: *Siccibacter* in T3 (9.7%), *Clavibacter* in T2 (10.7%), *Pseudomonas* and *Phytobacter* in T4 (9.3% and 6.4%, respectively).

*Pantoea* showed higher abundances in tomatoes grown under OF (2.5% and 4.2%, in T1 and T3, respectively).

*Chania*, *Cedecea*, *Azomonas*, and *Lactococcus* were recognized as major genera only in one experimental condition: *Chania* in T4 (3.9%), *Cedecea* in T2 (2.3%), *Azomonas* in T4 (1.3%), and *Lactococcus* in T3 (1.1%).

**Figure 4:**
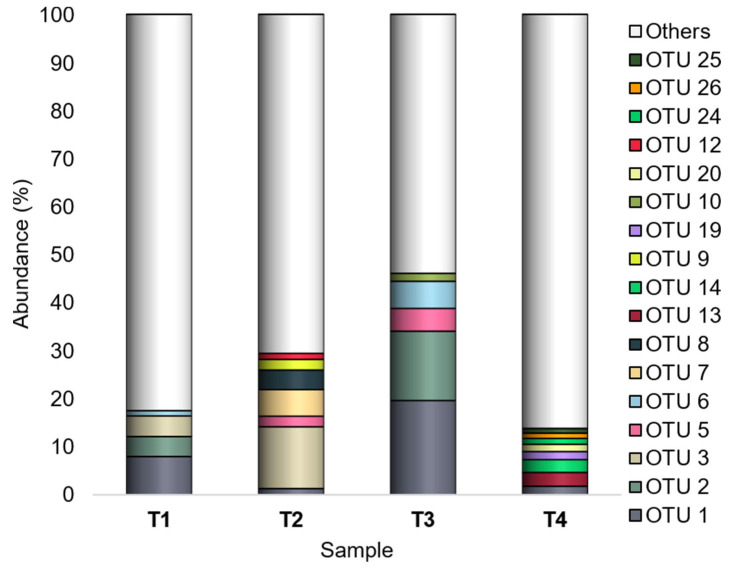
Bacterial composition at the OTU level. Distribution of the major OTUs (Ra ≥ 1% in at least one sample); OTUs with Ra < 1% were gathered in “Others”. T1: WT/OF, T2: WT/CF, T3: SB/OF, T4: SB/CF.

Among the 10,287 defined OTUs, the most abundant ones (having a number of mapping sequences > 1000 on the whole dataset) were OTU 1 (*Buttiauxella*), OTU 2 (order *Enterobacteriales*), OTU 3 (*Atlantibacter*), OTU 5 (*Acinetobacter*), OTU 6 (order *Bacillales*), OTU 7 (*Clavibacter*), and OTU 8 (family *Enterobacteriaceae*). Overall, OTU 1, OTU 2, and OTU 3 were the most represented (Figure 4). 

OTU 1 was the sole showing Ra ≥ 1% in all experimental conditions, and it was recorded with the highest abundances (7.9% and 19.6% in T1 and T3, respectively) in both tomato genotypes grown under OF. 

OTU 2 showed Ra < 1% in both tomato genotypes grown under CF, and it showed a peak (14.4%) in T3 (SB/OF). 

Different from OTU 1 and OTU 2, showing different abundances according to the farming condition, OTU 3 had higher abundances in WT than SB tomatoes. It was detected with Ra < 1% in SB tomatoes, and it showed a peak (12.9%) in T2 (WT/CF). 

Also OTU 6 revealed different abundances according to the farming condition, although with less marked differences than those observed for OTU 1 and OTU 2. It was detected with higher Ra_s_ in tomatoes grown under OF (1.1% and 5.6% in T1 and T3, respectively), whereas it was a rare OTU in those grown under CF.

OTU 5 did not reveal abundance differences according to the tomato genotype or the farming condition; it was a rare OTU in T1 and T4 and had Ra ≥ 1% in T2 (2.2%) and T3 (4.7%).

Finally, OTU 7 and OTU 8 had Ra ≥ 1% only in WT tomatoes grown under CF (OTU 7:5.5%; OTU 8:4.1%).

### 3.2. Unique and Shared Bacterial OTUs

The comparison of the peel-associated bacterial communities revealed a very low number of ubiquitous OTUs (common to all experimental conditions) and a high number of specific members (OTUs exclusively found in one experimental condition).

**Figure 5:**
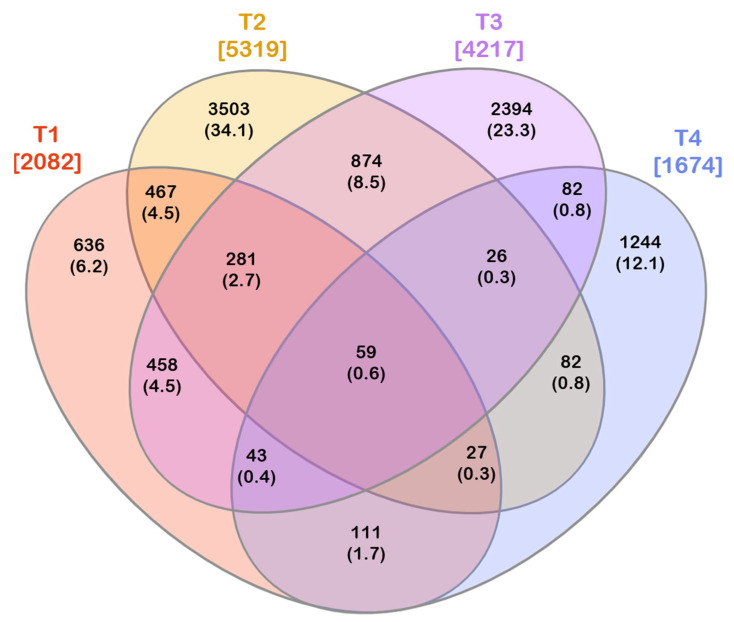
Venn diagram depicting the number of exclusive and shared OTUs among the investigated experimental conditions. The total number of OTUs found in each experimental condition is reported in square brackets. Both the number of exclusive/shared OTUs and the corresponding percentage (in round brackets) on the total of the OTUs are reported. T1: WT/OF, T2: WT/CF, T3: SB/OF, T4: SB/CF.

The four experimental conditions shared 59 out of 10,287 OTUs, representing the core microbiota (Figure 5). Except for OTU 7, which was shared only by three experimental conditions (T1, T2, and T3), the core microbiota included all the other most abundant OTUs.

**Figure 6:**
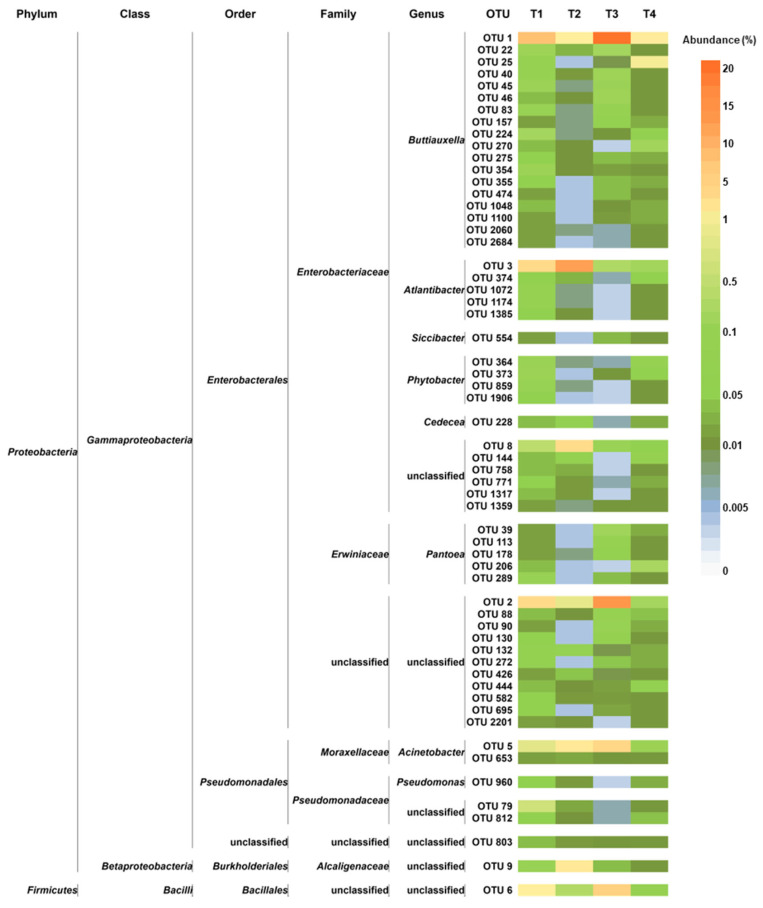
Distribution of the core microbiota OTUs across the studied experimental conditions. For each OTU, the taxonomic annotation is provided. T1: WT/OF, T2: WT/CF, T3: SB/OF, T4: SB/CF.

The core microbiota represented 22.6%, 24.5%, 46.9%, and 5.1% of the peel-associated bacterial communities in T1, T2, T3, and T4, respectively. It is worth noting that it accounted for a similar proportion in WT tomatoes grown under OF and CF, whereas it represented a very different moiety in communities found in SB, depending on the farming condition. Only one OTU belonged to *Firmicutes* (order *Bacillales*), whereas all the remaining OTUs belonged to *Proteobacteria* (Figure 6). Furthermore, only representatives of the following eight genera were found in the core microbiota: *Buttiauxella*, *Atlantibacter*, *Acinetobacter*, *Siccibacter*, *Pseudomonas*, *Phytobacter*, *Pantoea*, and *Cedecea*.

Differently from the core microbiota, including a low number of OTUs, a high number of exclusive OTUs were found in each experimental condition. The number of exclusive OTUs was 636, 3503, 2394, and 1244 for T1, T2, T3, and T4, respectively (Figure 5 Diagramma Venn OTU). 

Among the 2082 OTUs found in T1, 31% were exclusive to this experimental condition. These exclusive OTUs (having Ra_s_ in-between 0.04–0.26%) accounted for 30.6% of the peel epiphytic bacterial community in WT tomatoes grown under OF. These OTUs belonged to the phyla *Proteobacteria*, *Actinobacteria*, *Bacteroidetes*, *Firmicutes*, *Cyanobacteria*, *Verrucomicrobia*, and *Rhodothermaeota* (Table S1). Among these OTUs, 126 were classified at genus level; they belonged to 42 genera, with *Atlantibacter* and *Metakosakonia* as the most represented genera (including 26 and 13 OTUs, respectively) (Table S1).

In T2, 66% of the recorded OTUs were exclusive. These OTUs (with Ra_s_ in-between 0.008–0.31%) collectively accounted for 45.7% of the bacteria found on WT tomatoes grown under CF. Moreover, they were representatives of the phyla *Proteobacteria*, *Bacteroidetes*, *Actinobacteria*, *Firmicutes*, *Cyanobacteria*, *Tenericutes*, *Verrucomicrobia*, *Campilobacterota*, and *Deinococcus-Thermus* (Table S2). The OTUs classified at the genus level were representatives of 112 genera; in particular, *Atlantibacter* and *Clavibacter* (411 and 291 OTUs, respectively) were the most retrieved taxa (Table S2). 

Fifty-seven percent of the OTUs found in T3 were exclusive. These OTUs, showing Ra_s_ in the range 0.006–0.14%, accounted for 27.1% of the bacterial communities of SB tomatoes grown under OF. At the phylum level, these OTUs belonged to *Proteobacteria*, *Firmicutes*, *Actinobacteria*, *Rhodothermaeota, Bacteroidetes*, and *Cyanobacteria* (Table S3). In addition, the OTUs exclusively found in T3 were representatives of 22 genera; among them, *Siccibacter*, *Buttiauxella*, and *Pantoea* (344, 199, and 102 OTUs, respectively) were the most found (Table S3).

In T4, 74% of the recovered OTUs were exclusive. These OTUs (with Ra_s_ in-between 0.03–1.66%; among them, only three OTUs had Ra ≥ 1%) accounted for 71.6% of the peel epiphytic bacteria of SB tomatoes grown under CF. At the phylum level, these OTUs were ascribed to *Proteobacteria*, *Actinobacteria*, *Firmicutes*, *Bacteroidetes*, *Cyanobacteria*, *Tenericutes*, and *Verrucomicrobia* (Table S4). In addition, the OTUs classified at the genus level were ascribed to 63 genera; *Pseudomonas*, *Buttiauxella*, *Phytobacter*, *Chania*, and *Atlantibacter* (80, 71, 65, 55, and 51 OTUs, respectively) were the most abundant (Table S4).

### 3.3. Alpha- and Beta-Diversity Analyses

Alpha diversity analysis was assessed by the Shannon diversity and Pielou evenness indices. The Shannon index was 6.734, 6.821, 5.515, and 6.673 for T1, T2, T3, and T4, respectively. It significantly differed between SB tomatoes grown under OF and CF, whereas no differences were observed between WT tomatoes in relation to the farming condition (based on Solow test). Considering the farming conditions, Shannon index values significantly differed between WT and SB grown under OF, but not between WT and SB grown under CF (Solow test). The Pielou index was 0.881, 0.795, 0.661, and 0.899 for T1, T2, T3, and T4, respectively. According to the Solow test, significant differences in community evenness were revealed among all experimental conditions except for T1 (WT/OF) and T4 (SB/CF). Overall, alpha diversity indicated that the T3 (SB/OF) peel-associated bacterial communities showed the lowest diversity and evenness (lowest Shannon and Pielou index values), and therefore they were characterized by the presence of some dominant OTUs.

PCA and clustering analyses were performed to evaluate the effect of the tested conditions (genotype × farming condition) on the epiphytic bacterial communities found on the tomato peel.

**Figure 7:**
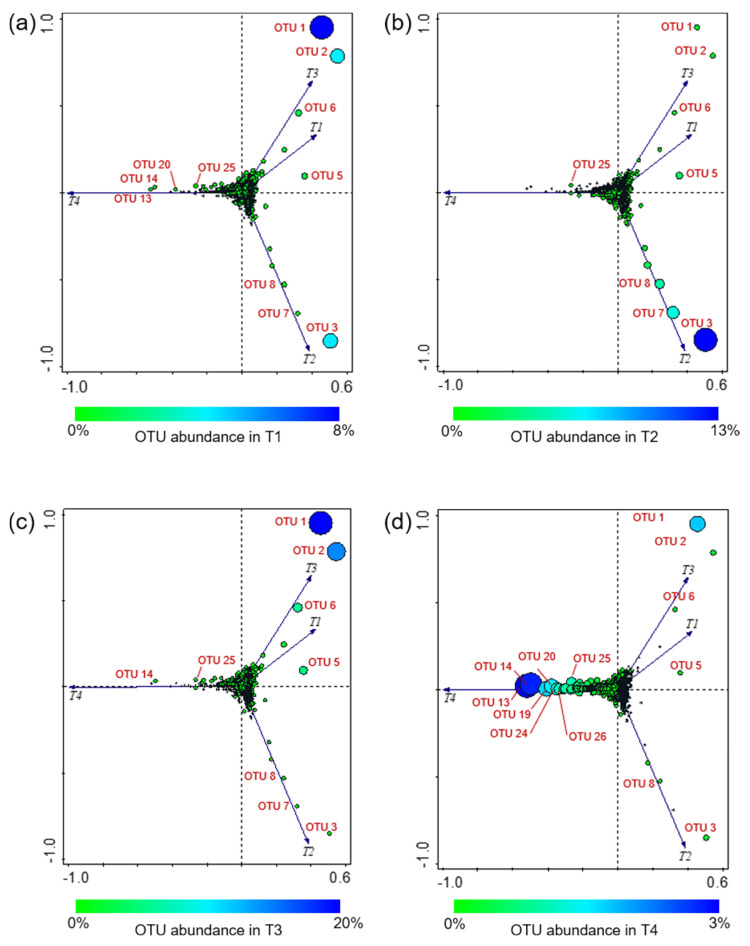
Principal component analysis (PCA) based on the bacterial OTU found across the investigated experimental conditions. Four plot visualizations of the same PCA analysis are provided to better show the OTU occurrence in the four experimental conditions; OTU abundance variation in T1 (**a**), T2 (**b**), T3 (**c**), and T4 (**d**). Circle size is proportional to the OTU relative abundance in the samples; OTU absence in an experimental condition is indicated by “+”. T1: WT/OF, T2: WT/CF, T3: SB/OF, T4: SB/CF.

Four plot visualizations were provided for the same PCA analysis to better show the OTU occurrence variation according to the four experimental conditions (Figure 7).

Overall, the PCA (explaining 75.87% of the total variance) showed that T1 and T3 grouped together, indicating that the bacterial communities of both tomato genotypes grown under OF were similar. Conversely, T2 and T4 were separated from each other and from the other conditions. 

As an overview, it is possible to observe a cloud of OTUs at the axis intersection, indicating a cluster of OTUs showing no notable differences (in distribution and abundance) among the various experimental conditions. By contrast, some OTUs, well separated from the cluster, appear to be much more oriented toward a certain condition. In particular, OTU 1, OTU 2, OTU 5, and OTU 6 were close to T1 and T3 (Figure 7a,c). As reported above, OTU 1, OTU 2, and OTU 6 showed different abundances according to the farming condition, having higher abundances in both tomato genotypes grown under OF; OTU 5 was detected with the highest abundance in T3. OTU 3, OTU 7, and OTU 8 were closer to T2, showing their highest abundances in WT tomatoes grown under CF (Figure 7b). OTU 13, OTU 14, OTU 19, OTU 20, OTU 24, OTU 25, and OTU 26 were ordered along the T4 vector (Figure 7d); these OTUs were detected with Ra_s_ in-between 1–3% only in SB grown under CF, whereas they were rare OTUs in all other conditions.

**Figure 8:**
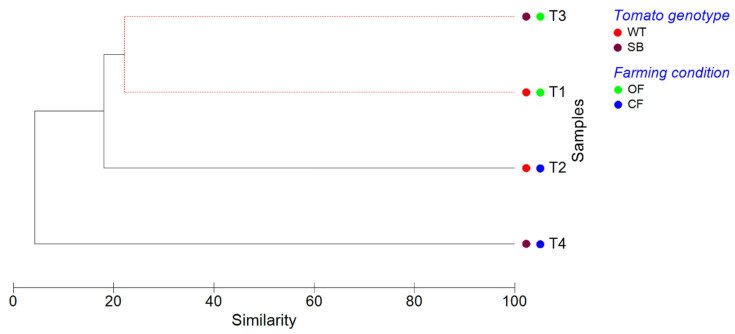
Cluster analysis dendrogram showing similarities/dissimilarities among the experimental conditions based on their OTU composition. The clustering analysis (complete linkage) was performed on Bray–Curtis dissimilarity data, applying SIMPROF routine to test significance. Solid black lines indicate samples that are significantly different (*p* < 0.05), whereas dashed red lines indicate groups of samples that are not significantly different (*p* > 0.05), according to SIMPROF test.

The clustering analysis (Figure 8) confirmed that the bacterial communities found in T1 and T3 did not show significant differences (*p* < 0.05, SIMPROF test). In addition, the hierarchical clustering dendrogram showed that T4 communities were the most dissimilar.

## 4. Discussion

The selection of tomato cultivars with a high content of antioxidants is one of the research trends in the production of nutritionally superior genotypes. In this context, no investigation has been carried out to compare the microbiota of red and purple tomatoes, and to get a more extended overview of the possible bacterial assemblage differences of the two genotypes subjected to different farming practices.

The current study sought to provide the first information on this topic, comparing the peel-associated epiphytic bacteria of the WT (red) and SB (purple) tomatoes grown under organic and conventional farming systems.

The taxonomic profiling showed that *Proteobacteria*, *Firmicutes*, *Actinobacteria*, and *Bacteroidetes* were the major taxa across all experimental conditions, with *Proteobacteria* as the dominant phylum. These taxa are ubiquitous in the soil and are widely found on the surface of fruits and vegetables, including tomatoes [44,45,46]. 

*Enterobacteriaceae*, *Pseudomonadaceae*, *Moraxellaceae*, *Microbacteriaceae*, *Erwiniaceae*, *Yersiniaceae*, *Rhodobacteraceae*, *Alcaligenaceae*, *Cytophagaceae*, *Weeksellaceae*, *Streptococcaceae*, and *Oceanospirillaceae* were detected as major families. Most of these families or some of their genera are commonly associated with edible plants and are recorded in both epiphytic and endophytic bacterial communities [44,47,48]. 

It is worth noting that in this work most of the peel-associated bacteria found across all the experimental conditions belonged to the family *Enterobacteriaceae.* These results are consistent with those reported in the literature. *Enterobacteriaceae* is among the most represented families in both epiphytic and endophytic plant microbiota [44,49]. Many members are natural inhabitants of several food plants, and a high abundance of this taxon on the surface of various fruits and vegetables (including tomatoes) has been reported [28,44,46,50]. In particular, a high *Enterobacteriaceae* prevalence is a feature of the bacterial communities of fruits and vegetables grown close to the soil (e.g., strawberries, lettuce, peppers, and tomatoes) [28,44]. 

Among the 161 annotated genera, *Buttiauxella* and *Atlantibacter* were the most abundant (with 11,005 and 7156 sequences over the whole dataset, respectively), and they showed an opposite pattern of prevalence according to the tomato genotype. *Buttiauxella* had the highest abundances in SB tomatoes, in particular in those grown under CF (Ra = 27%); *Atlantibacter* was mainly found in WT tomatoes and showed the highest abundance (23.3%) in those grown under CF. 

*Buttiauxella* species have been isolated especially from the intestines of snails and slugs, but have been also retrieved in water and soil [51,52]. Various *Buttiauxella* members have been found in the plant rhizosphere, where they can play important roles as phosphate solubilizers and plant growth promoters [53,54,55]. Some members have been found in plant phyllosphere [56], including that of tomato [57,58]. 

*Atlantibacter* has been proposed as a novel genus to include the two species *A. hermannii* and *A. subterranea* (formerly *Escherichia hermannii* and *Salmonella subterranea*) [59]. *A. hermannii* members have clinical significance, being mostly found in human wounds, sputum, and stool samples [60], whereas *A. subterranea* strains were isolated from a river and heavy metal contaminated sediments [61,62]. Scarce information is available on *Atlantibacter* members found in association with plants and their possible roles. However, some of them were revealed among plant microbiotas (e.g., in tobacco leaves, maize aerial root mucilage, and wheat bran) [63,64,65], and a representative of this genus (*A. hermannii* DDE1) featuring potential plant growth-promoting ability was found in pumpkin roots [66].

The other major genera that we detected on the tomato peels were *Acinetobacter*, *Siccibacter*, *Clavibacter*, *Pseudomonas*, *Phytobacter*, *Pantoea*, *Chania*, *Cedecea*, *Azomonas,* and *Lactococcus*. These taxa have members that are common plant and/or soil residents [67,68,69,70,71,72,73,74,75], mostly featuring beneficial activities toward the plants [74,75,76,77]. However, some of these genera include also detrimental bacteria [78,79].

*Acinetobacter*, *Pseudomonas*, and *Pantoea* are commonly found in high abundance in the rhizosphere and phyllosphere (including carposphere) of various plants, including tomato [28,46,55,58,65,80]. Various strains of these taxa have shown beneficial interactions with various plants (including tomato), owing to their abilities as plant growth promoters, bio-fertilizers, and biocontrol agents [22,74,77,81]. As for *Pseudomonas* and *Pantoea*, although encompassing a great number of beneficial members (even those having antagonistic activity toward tomato phytopathogens [81,82,83,84]), they include also plant pathogens. Notable examples are *Pseudomonas syringae* and *Pantoea ananatis*, which cause diseases in various economically important crops, including the bacterial speck and graywall of tomato [78,85].

The current study evidenced that *Pantoea* and *Pseudomonas* showed a different distribution according to the farming condition, with an opposite pattern of prevalence: *Pantoea* had the highest abundances (2.5–4.2%) in tomatoes grown under OF, whereas *Pseudomonas* was a minor taxon (Ra_s_ in-between 0.02–0.7%) in OF tomatoes and a major genus in CF tomatoes, showing a peak of abundance (9.3%) in SB/CF. However, it is not possible to discriminate possible beneficial/detrimental representatives among the OTUs ascribed to these genera, since the length of the sequenced fragment does not allow to gain deeper insight. Differently, *Acinetobacter* occurrence on tomato peel seemed not to be related to the tomato genotype or farming system, being present in all the experimental conditions as a major taxon and showing the highest abundances in T2 (WT/CF) and T3 (SB/OF). 

Another taxon deserving discussion is *Clavibacter*. It is generally recognized as a genus of phytopathogens of agricultural significance, including those involved in the bacterial canker of tomato [86]. Nevertheless, the occurrence of tomato non-pathogenic *Clavibacter* strains has been reported [71].

Our results showed that *Clavibacter* had a different distribution according to the tomato genotype, being a rare taxon (Ra = 0.1%) in SB and a major genus in WT tomatoes, with an abundance peak (10.7%) in those grown under CF. However, since the tomatoes collected in this study did not show bacterial canker lesions, it is possible to assume that the detected peel-associated *Clavibacter* OTUs were non-pathogenic representatives.

In the current investigation, 10,287 OTUs were recognized across the whole dataset. Our results evidenced that a high number of exclusive OTUs were found in each experimental condition, but they showed a very low abundance. Differently, the tomato peel-associated core microbiota consisted of a much lower OTU number (59 OTUs, representing ~0.6% of the total number of OTUs). It is worth noting that the core microbiota accounted for a similar proportion in WT tomatoes grown under OF and CF (22.6% and 24.5%, respectively), whereas it represented a very different moiety in SB communities, depending on the farming system. Among the communities related to the various experimental conditions, the core microbiota represented the highest proportion in those retrieved on SB grown under OF (46.9%) and the lowest fraction (accounting for 5.1% of the community only) in those of SB grown under CF.

Overall, the core microbiota OTUs were representative of eight genera only: *Buttiauxella*, *Atlantibacter*, *Acinetobacter*, *Siccibacter*, *Pseudomonas*, *Phytobacter*, *Pantoea*, and *Cedecea*. As discussed above, these taxa include various beneficial members.

Looking deeper at the shared OTUs, it is possible to note that among them there were almost all the most abundant OTUs (the exception was OTU 7, being absent in T4), suggesting a possible key role in the structuring and preservation of tomato peel communities. 

However, the most abundant OTUs occurrence was different among the investigated conditions. The PCA analysis evidenced that OTU 1 (*Buttiauxella*), OTU 2 (order *Enterobacteriales*), OTU 5 (*Acinetobacter*), and OTU 6 (order *Bacillales*) were related to T1 and T3 (WT/OF and SB/OF, respectively) (Figure 7a,c). Actually, OTU 1, OTU 2, and OTU 6 showed different abundances according to the farming condition, having higher abundances in tomatoes (both genotypes) grown under OF (Figure 4). Instead, OTU 5 was detected with the highest abundance in WT/OF condition (T3) (Figure 4). OTU 3 (*Atlantibacter*), OTU 7 (*Clavibacter*), and OTU 8 (family *Enterobacteriaceae*) were related to T2 (Figure 7b), showing their highest abundances in WT tomatoes grown under CF (Figure 4). In particular, OTU 3 was a rare OTU in SB tomatoes and among the major OTUs in WT tomatoes, showing an abundance peak (12.9%) in WT/CF condition. OTU 7 and OTU 8 represented rare OTUs in almost all experimental conditions, except in WT tomatoes grown under CF. 

Alpha diversity analysis indicated that the farming system affected the peel-associated bacterial community diversity of SB tomatoes, being significantly lower in SB grown under OF than those grown under CF. Conversely, no significant differences in the community diversity were observed in WT tomatoes according to the farming condition. Considering the same farming condition, significant differences in bacterial diversity between the two different genotypes were observed only for the organic crop management. The bacterial communities of WT/OF tomatoes showed higher diversity than those of SB/OF. Both genotype and farming conditions seemed to affect bacterial evenness. Significant differences were revealed in the community evenness of both WT and SB tomatoes according to the farming condition. The bacterial communities of WT grown under OF showed a higher evenness than those grown under CF. On the contrary, the bacterial communities of SB grown under OF showed a lower evenness than those grown under CF. Considering the same farming condition, significant differences in bacterial evenness between the two different genotypes were observed for both crop managements. In OF, the bacterial communities of SB tomatoes showed a lower evenness than those of WT. By contrast, in CF, the bacterial communities of SB tomatoes were characterized by a higher evenness than those of WT. 

Overall, the PCA analysis showed that T1 and T3 grouped together, indicating that the bacterial communities of both tomato genotypes grown under OF were similar. On the contrary, T2 and T4 were well separated, indicating that the bacterial communities found on WT and SB tomatoes grown under CF differed from each other and from those of the other experimental conditions. This was confirmed by the clustering analysis (Figure 8), revealing that the bacterial communities of T1 and T3 did not show significant differences (*p* < 0.05, SIMPROF test).

## 5. Conclusions

To the best of our knowledge, this work was the first investigation regarding the peel-associated epiphytic bacteria of red and purple (anthocyanin-rich) tomatoes subjected to different farming systems. This study provided the first insights into similarities/differences among the bacterial assemblages revealed in both genotypes grown under organic and conventional farming. Further investigations would be useful to give a more extended view of the tomatoes-bacteria associations, to provide a comparative analysis of the bacterial assemblages in other compartments of the tomato plant (in particular in pulp, surveying also the possible presence of bacteria with implications for human health). In addition, it would be interesting to investigate if new bacterial strains with potential beneficial effects for the plant (to be also employed in sustainable agriculture) are present and/or enriched in the purple tomatoes. Moreover, future developments of the study could involve additional factors, such as different soils and/or locations for the farming to address possible effects of biogeography on the microbiota.

## Data Availability

The sequence data are available at the Sequence Read Archive (SRA; https://www.ncbi.nlm.nih.gov/sra, accessed on 1 October 2022) repository; study accession number: PRJNA798869.

## Supplementary Materials

The following supporting information can be downloaded at: https://www.mdpi.com/article/10.3390/microorganisms10112240/s1, Table S1: OTUs exclusively found in T1 (WT/OF); Table S2: OTUs exclusively found in T2 (WT/CF); Table S3: OTUs exclusively found in T3 (SB/OF); Table S4: OTUs exclusively found in T4 (SB/CF).
